# Efficient production of the β-ionone aroma compound from organic waste hydrolysates using an engineered *Yarrowia lipolytica* strain

**DOI:** 10.3389/fmicb.2022.960558

**Published:** 2022-09-21

**Authors:** Shuyi Chen, Yanping Lu, Wen Wang, Yunzi Hu, Jufang Wang, Shixing Tang, Carol Sze Ki Lin, Xiaofeng Yang

**Affiliations:** ^1^Guangdong Provincial Key Laboratory of Tropical Disease Research, School of Public Health, Southern Medical University, Guangzhou, Guangdong, China; ^2^School of Biology and Biological Engineering, South China University of Technology, Guangzhou, Guangdong, China; ^3^Technology Research Center, Wuliangye Yibin Company Limited, Yibin, Sichuan, China; ^4^Postdoctoral Research Workstation, Sichuan Yibin Wuliangye Group Company Limited, Yibin, Sichuan, China; ^5^Guangzhou Institute of Energy Conversion, Chinese Academy of Sciences (CAS), Guangzhou, Guangdong, China; ^6^School of Energy and Environment, City University of Hong Kong, Kowloon, Hong Kong SAR, China

**Keywords:** metabolic engineering, *Yarrowia lipolytica*, CCD1, β-ionone, flavor and fragrance compound, food and agricultural waste

## Abstract

This study demonstrates the feasibility of establishing a natural compound supply chain in a biorefinery. The process starts with the biological or chemical hydrolysis of food and agricultural waste into simple and fermentative sugars, followed by their fermentation into more complex molecules. The yeast strain, *Yarrowia lipolytica*, was modified by introducing high membrane affinity variants of the carotenoid cleavage dioxygenase enzyme, PhCCD1, to increase the production of the aroma compound, β-ionone. The initial hydrolysis process converted food waste or sugarcane bagasse into nutrient-rich hydrolysates containing 78.4 g/L glucose and 8.3 g/L fructose, or 34.7 g/L glucose and 20.1 g/L xylose, respectively. During the next step, engineered *Y. lipolytica* strains were used to produce β-ionone from these feedstocks. The yeast strain YLBI3120, carrying a modified *PhCCD1* gene was able to produce 4 g/L of β-ionone with a productivity of 13.9 mg/L/h from food waste hydrolysate. This is the highest yield reported for the fermentation of this compound to date. The integrated process described in this study could be scaled up to achieve economical large-scale conversion of inedible food and agricultural waste into valuable aroma compounds for a wide range of potential applications.

## Introduction

While the climate change and the COVID-19 pandemic are exacerbating the global supply chain disruption, a significant amount of organic waste is being deposited in landfills (Papargyropoulou et al., 2014; Goldthau and Hughes, 2020; Falkendal et al., 2021; Lo et al., 2021). It is estimated that one third of food intended for human consumption, 1.3 billion tons annually, is lost or wasted (Gustavsson et al., 2011; Kummu et al., 2012). In addition, most waste generated by the food processing industry contains lignocellulose, such as sugarcane bagasse, wheat straw, and cornstalk (Ravindran and Jaiswal, 2016). These waste products represent the most abundant renewable carbon resource in the world (Saini et al., 2015; Cho et al., 2020). Strategies integrating organic waste management and biorefinery processes could facilitate nutrient recovery and the production of chemicals, potentially stabilizing global food-supply chains (Lin et al., 2013; Xiong et al., 2019; Leong et al., 2021).

Plant-derived terpenoid flavor and fragrance compounds are widely used in the aroma industry, with demand rapidly increasing (Schempp et al., 2018; Chen et al., 2020). However, low yields and high costs limit the viability of directly extracting terpenoids from plants and other naturally occurring sources (Liu and Nielsen, 2019). Converting natural raw materials into aroma compounds utilizing microorganisms generally recognized as safe (GRAS) represents a promising approach for the production of aroma compounds from renewable biomass (Berger and Zorn, 2007).

Genetically engineering microbial cells for the biosynthesis of flavor and fragrance compounds is an emerging sustainable alternative to traditional chemical synthesis or plant-based extraction (Schempp et al., 2018; Chen et al., 2020). β-ionone is an aroma compound that is widely used in the food and cosmetic industries. It is also a key intermediate in the production of vitamins A, E and K (Lalko et al., 2007; Gonzalez-Verdejo et al., 2015; Paparella et al., 2021). The molecule is an apocarotenoid that is derived from β-carotene, a C40 terpenoid formed by carotenoid cleavage dioxygenases (CCDs) (Simkin et al., 2004; Werner et al., 2019).

Microbial production of β-ionone was first demonstrated in *Saccharomyces cerevisiae* following the introduction of the β-carotene biosynthesis pathway from *Xanthophyllomyces dendrorhous* and the raspberry *RiCCD1* gene (Beekwilder et al., 2014). Other microbes, such as *Escherichia coli* (Zhang et al., 2018) and *Yarrowia lipolytica* (Czajka et al., 2018; Lu et al., 2020) have been engineered to heterologously biosynthesize β-ionone. In these systems, the highest reported β-ionone yields were 0.5 g/L in *E. coli* (Zhang et al., 2018), 0.4 g/L in *S. cerevisiae* (Simkin et al., 2004; Werner et al., 2019), and 1.0 g/L in *Y. lipolytica* (Lu et al., 2020).

*Y. lipolytica* is a GRAS yeast that, due to its endogenous mevalonate pathway (MVA), high capacity to generate acetyl coenzyme A, efficient lipid metabolism, and wide substrate scope, is rapidly emerging as a promising tool for the production of terpenoids (Ma et al., 2019; Li et al., 2021; Zhang et al., 2021). Metabolic engineering strategies and synthetic biology tools raise the possibility of redirecting microbial carbon fluxes in *Y. lipolytica* to create efficient cellular pathways for the production of natural compounds (Schwartz et al., 2016; Liu et al., 2022). Using a modular approach, we previously engineered the *Y. lipolytica* strain YLBI3118, which was able to produce β-ionone at a titer of 1.0 g/L in a 3-L fermenter using fed-batch fermentation. In those experiments, we also noted that a significant proportion of β-carotene was not converted into β-ionone (Lu et al., 2020).

Recent studies in the literature pointed to potential solutions to overcome the limited efficiency of the process. (1) The improved membrane affinity of the *Petunia hybrida* CCD enzyme (PhCCD1) was found to enhance the conversion of β-carotene into β-ionone in *S. cerevisiae* (Werner et al., 2019). (2) Reports supporting the use of *Y. lipolytica* for the production of high value-added chemicals from food waste (Gao C. et al., 2016; Li et al., 2022; Lopes et al., 2022). Based on these observations, we introduced the modified *PhCCD1* gene into the YLBI3118 strain. As demonstrated here, the resulting *Y. lipolytica* strains produced β-ionone at an improved yield from food waste and sugarcane bagasse. This work describes the integrated process of converting organic waste streams into valuable commercial flavors and fragrances.

## Materials and methods

### Strains, media, and culture conditions

*E. coli* DH5α was used for cloning and plasmid construction. It was cultured in lysogeny broth (10 g/L of tryptone, 5 g/L of yeast extract and 10 g/L of sodium chloride) supplemented with 50 mg/mL of ampicillin at 37°C under constant shaking of 250 rpm. *Y. lipolytica* strains were cultured in modified yeast extract peptone dextrose (YPD) medium (YPDm; 10 g/L of yeast extract, 20 g/L of tryptone and 20 g/L of glucose) or organic waste hydrolysate. A synthetic dextrose medium containing 6.7 g/L of yeast nitrogen base, 0.62 g/L of drop-out supplement lacking leucine, 20 g/L of glucose, and 5 g/L of ammonium sulfate was used for the selection of strains carrying the introduced biochemical pathway. Yeast strains were incubated with shaking (250 rpm) at 30°C. For plate cultivation, 2% agar was added to the culture medium. For β-ionone fermentation, yeast strains were incubated in 250-mL flasks containing 25 mL of YPDm medium and 10% (v/v) dodecane with shaking (250 rpm) at 20°C.

### Construction and verification of engineered *Y. lipolytica* strains

Plasmids used in this study are listed in Table 1, while primers used for the construction of these plasmids are listed in Supplementary Table 1. The original clustered regularly interspaced short palindromic repeats (CRISPR)-Cas9-mediated genome editing plasmid, pCAS1yl, was a kind gift from Prof. Sheng Yang (Gao S. et al., 2016). The 20-nucleotide sequence at the 5′-end of the guide RNA was modified for gene unit integration using the oligonucleotides listed in Supplementary Table 1. The *PhCCD1* expression cassette was constructed as described previously (Lu et al., 2020). Site directed mutagenesis or the amino-terminal addition of membrane insertion peptide was performed by overlap-PCR. The selection marker *Leu2*, amplified from pINA1269, was used to monitor gene unit integration. The DNA fragments were assembled by Gibson assembly (Gibson et al., 2009). The created plasmids were linearized by *Not*I digestion. The transformation of the linear plasmid DNA fragments into *Y. lipolytica* was performed using the Frozen-EZ Yeast Transformation II Kit (Zymo Research Corporation, Orange, CA, United States). The transformation mix was spread onto a selection plate and cultured at 30°C for 4 days. Details on the plasmid construction are given in the Supplementary File.

**TABLE 1:** Plasmids used in this work.

Plasmids	Characteristics	Source
pCAS1yl-POX4	*POX4* guide RNA module and Cas9 expression cassette in pMCSCen1	This work
pUC19-POX4-HA	Up/downstream of *POX4* locus in pUC19	This work
pUC19-POX4-Leu2	*Leu2* gene expression cassette in pUC19-POX4-HA	This work
pUC19-CCD1m	*P_GPD2_-CCD1 (K164L)-mig1t* in pUC19-POX4-Leu2	This work
pUC19-lck-CCD1	*P_GPD2_-lck-CCD1-mig1t* in pUC19-POX4-Leu2	This work
pUC19-lck-CCD1m	*P_GPD2_-lck-CCD1 (K164L)-mig1t* in pUC19-POX4-Leu2	This work

The previously constructed YLBI3118 strain (Lu et al., 2020) was used as the parent strain in the current study. The acyl-coenzyme A oxidase 4 (*POX4*) locus was selected as the site for gene integration. One of three integration cassettes [PhCCD1 (K164L), lck-PhCCD1, and lck-PhCCD1 (K164L)] was integrated into the *POX4* locus of the YLBI3118 strain to generate the YLBI3120, YLBI3121, and YLBI3122 strains. Yeast strains used in this study are listed in Table 2. Colonies were PCR screened for their genotype using the KOD FX DNA Polymerase (Toyobo, Osaka, Japan) and the primers listed in the Supplementary Table 2. To verify the genetic stability of the strains, three colonies of YLBI3120, YLBI3121, and YLBI3122 were cultivated in YPDm medium for 12-days and re-screened using the same PCR conditions used for plasmid construction with the primers listed in Supplementary Table 2. The PCR products were sequenced at Sangon Biotech Co., Ltd. (Shanghai, China).

**TABLE 2:** *Y. lipolytica* strains used in this work.

Strains	Characteristics	Source
YLBI3118	A β-ionone-producing strain engineered from the Po1f strain	Lu et al., 2020
YLBI3119	YLBI3118 *POX4*::*Leu2* expressed cassette	Lu et al., 2020
YLBI3120	YLBI3118, *POX4*::*Leu2-P_GPD2_-CCD1 (K164L)-mig1t*	This work
YLBI3121	YLBI3118, *POX4*::*Leu2-P_GPD2_-lck-CCD1-mig1t*	This work
YLBI3122	YLBI3118, *POX4*::*Leu2-P_GPD2_-lck-CCD1 (K164L)-mig1t*	This work

### Biological or chemical hydrolysis of organic waste

Bakery waste, 1–2 days out of date, was collected from a Starbucks^§^ outlet in Shatin in Hong Kong. This bakery waste, comprised of bread (70–80%), pastries, and cakes (20–30%) was processed as described previously (Yang et al., 2015). Briefly, the waste was immediately homogenized using a domestic kitchen blender for 5 min, and the resulting homogenate was subjected to fungal enzymatic hydrolysis in a 2.5-L bioreactor (BioFlo/CelliGen 115, New Brunswick Scientific, Edison, NJ, United States). The blended bakery waste with a solid-to-liquid ratio of 42% (w/v) was combined with 1 L of water in the bioreactor. To initiate hydrolysis, this mixture was treated with two dishes of fungal mash (*Aspergillus awamori* and *A. oryzae*) obtained from solid state fermentation. The resulting mixture was stirred at 300 rpm at 55°C for 12 h. The resulting hydrolysate broth was centrifuged at 11,500 *g* for 30 min, after which the supernatant was subjected to successive vacuum filtration steps through a Whatman No. 1 filter paper and sterilized using a 0.22-μm membrane. The resulting solution was analyzed to determine its sugar and free amino nitrogen concentrations and stored at −20°C before use.

The enzymatic hydrolysis of sugarcane bagasse was performed as described previously (Wang et al., 2020). Briefly, sugarcane bagasse (particle size ≥ 0.42 mm) from Lianfeng Deep Processing of Agricultural Products (Jiangsu, China) was treated with 2% (w/v) sodium hydroxide to give a 10% solid–liquid ratio (w/v) mixture, and incubated at 80°C for 2 h. This mixture was immediately subjected to enzymatic hydrolysis by the addition of 20 FPU cellulase/g cellulose and 2.5 μL Tween 80/mL slurry. This mixture was stirred at 150 rpm at 50°C for 120 h. Particle free enzymatic hydrolysate was collected by centrifugation at 13,000 *g* for 30 min before fermentation.

### β-ionone production from organic waste hydrolysates

The fermentation in YPDm medium was performed as described (Lu et al., 2020). The fermentation of organic waste hydrolysate was performed in 250-mL flasks. The media contained either a onefold-diluted (0.5×) or undiluted (1×) organic waste hydrolysate supplemented with 5 g/L of yeast extract dissolved in 20 mM of phosphate buffer (pH 7.0). The *Y. lipolytica* seed culture was incubated in YPDm medium with shaking at 250 rpm at 30°C for 16 h. The resulting seed culture was inoculated at an initial 600-nm optical density (OD_600_) of ∼0.2 into 25 mL of hydrolysate medium containing 10% (v/v) dodecane. These cultures were incubated with shaking at 250 rpm at 20°C for 12 days. Samples were taken for OD_600_ measurement, high-performance liquid chromatography (HPLC), and gas chromatography (GC) analyses. Experiments were conducted in triplicates.

### Biomass and sugar quantification

A 1-mL culture aliquot was harvested by centrifuging (5,000 *g* for 10 min), washed in water once, and dried at 60°C for 48 h. The resulting dry material was weighed to obtain the dry cell weight (DCW). The glucose, fructose, xylose, arabinose, and cellobiose content of the cells were analyzed by HPLC on an Agilent 1260 Infinity series system (Agilent Technologies, Santa Clara, CA, United States) equipped with an Aminex HPX-87P column (300 mm × 7.8 mm, Bio-Rad, Hercules, CA, United States) and a refractive index detector (Lai et al., 2014). Analytes were eluted with 1 mM of sulfuric acid at a flow rate of 0.6 mL/min, with the column temperature set to 60°C and the detector temperature set to 35°C. All samples were passed through a 0.22-μm filter before HPLC analysis.

### β-ionone quantification

β-ionone in the aqueous medium and cell pellets was extracted using dodecane as previously described (Czajka et al., 2018; Lu et al., 2020) and quantified by GC. Briefly, the supernatant (organic phase) was diluted appropriately and passed through a 0.22-μm pore-size membrane, and a 1-μL sample of the resulting filtrate was injected into a GC system (HP 7890A, Agilent Technologies) equipped with a DB-FFAP capillary column [60 m × 0.25 mm (internal diameter), 0.25-μm film thickness; J&W Scientific, Agilent Technologies, United States] coupled to a flame ionization detector. The column was first maintained at 80°C for 1 min; heated at 10°C/min to 120°C, and maintained at this temperature for 1 min; and finally heated at 10°C/min to 240°C. A standard curve was constructed for 1–100 mg/L β-ionone. Isolongifolene (Sigma-Aldrich, United States) was used as an internal standard.

### β-carotene quantification

β-carotene was quantified using a method described previously (Lu et al., 2020). Briefly, a 0.1-mL aliquot of cultured cells was harvested by centrifugation for 5 min at 13,000 *g*, re-suspended in 0.7-mL dimethyl sulfoxide, and incubated at 55°C for 10 min in a water bath. The resulting mixture was mixed with 0.7 mL of acetone, incubated at 45°C for 15 min, and centrifuged at 13,000 *g* for 5 min. The supernatant was passed through a 0.22-μm pore-size membrane and analyzed by HPLC on an Agilent 1260 Infinity Series system (Agilent Technologies, United States) equipped with an SB-C18 column (5 μm, 4.6 × 150 mm^2^, Agilent Technologies) and an ultraviolet light detector (wavelength 450 nm). The column was eluted with a mobile phase consisting of methanol, acetonitrile, and dichloromethane (42:42:16) at a flow rate of 1.0 mL/min, at a column temperature of 30°C.

## Results

### Improving β-ionone biosynthesis by the introduction of a *PhCCD1* variant into YLBI3118

**FIGURE 1:**
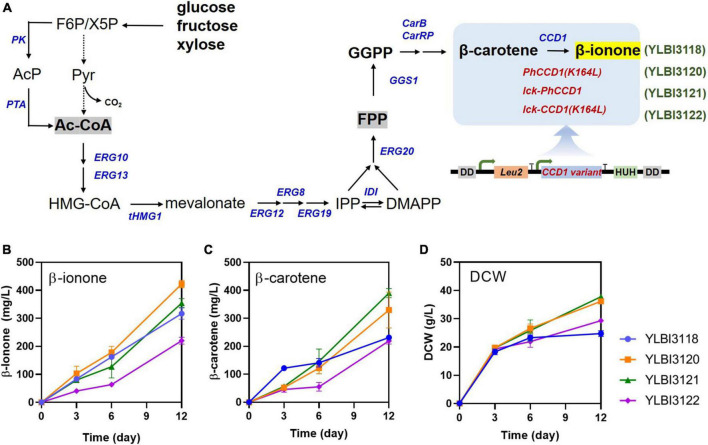
Metabolic engineering of *Y. lipolytica* for the highly efficient production of β-ionone. **(A)** The schematic diagram of the β-ionone biosynthesis pathway in engineered *Y. lipolytica*. **(B)** β-ionone, **(C)** β-carotene, and **(D)** biomass production (dry cell weight, DCW) at various time-points. Fermentation was conducted in 25 mL of YPDm medium containing 10% (v/v) dodecane. Cultures were incubated with shaking at 250 rpm at 20°C for 12 days. Each experiment was performed in triplicates. Abbreviations used in panel **(A)**: PK, phosphoketolase; PTA, phosphotransacetylase; ERG10, acetoacetyl-CoA thiolase; ERG13, hydroxymethylglutaryl-CoA synthase; tHMGR, truncated hydroxymethylglutaryl-CoA reductase; ERG8, phosphomevalonate kinase; ERG12, mevalonate kinase; ERG19, mevalonate diphosphate decarboxylase; IDI, isopentenyl diphosphate isomerase; ERG20, geranyl/farnesyl diphosphate synthase; GGS1, GGPP synthase; carB, phytoene dehydrogenase; carRP, phytoene synthase/lycopene cyclase; CCD1, carotenoid cleavage dioxygenase.

To increase β-ionone yield from *Y. lipolytica*, we first modified the YLBI3118 strain to enhance β-carotene cleavage. To achieve this, we introduced three different variants of the *PhCCD1* gene with high membrane affinity: *PhCCD1* (K164L), lck-*PhCCD1* and lck-*PhCCD1* (K164L). *PhCCD1* (K164L) contained a single amino acid residue substitution, a leucine-for-lysine substitution at position 164. The lck-*PhCCD*1 variant had a decapeptide derived from the *N*-terminal region of rat lymphocyte protein tyrosine kinase (lck) fused to its *N*-terminal. Both of these variants of *PhCCD1* were described previously (Werner et al., 2019). An additional chimeric lck-*PhCCD1* (K164L) was created by combining the single amino acid substituted K164L variant of the enzyme with the lck peptide tag. All constructs were placed under the control of the glycerol-3-phosphate dehydrogenase *promoter* (PGPD2), a strong constitutive promoter. Subsequently, each *PhCCD1* expression cassette was assembled with a native 3-isopropylmalate dehydrogenase gene (*Leu2*) expression cassette, and the resulting constructs were inserted into the *POX4* locus of the YLBI3118 strain using the CRISPR/Cas9 tool (Gao S. et al., 2016). Three yeast strains were generated in these experiments: the YLBI3120 strain incorporated the *PhCCD1 (K164L)* construct, the YLBI3121 strain incorporated *lck-PhCCD1* fusion protein, and the YLBI3122 strain carried the *lck-PhCCD1 (K164L)* construct (Figure 1A).

To investigate their ability to synthesize β-ionone, the YLBI3120, YLBI3121, and YLBI3122 strains were cultured in flasks containing YPDm medium with 10% (v/v) dodecane at 20°C for 12 days. We tested three transformants for each strain and observed a similar β-ionone production (coefficient of variation < 15%), data not shown. The best performing colonies were selected for further studies.

As shown in Figure 1B, the β-ionone concentration in the YLBI3120, YLBI3121, and YLBI3122 cultures were 422 ± 13 mg/L, 354 ± 16 mg/L, and 220 ± 12 mg/L, respectively. These figures equated to a 133, 112, and 69% of β-ionone production of the parent strain (YLBI3118). Thus, the introduction of both the *PhCCD1* (*K164L*) or *lck-PhCCD1* constructs resulted in an improved β-ionone yield.

In addition, the YLBI3120, YLBI3121 and YLBI3122 cultures contained 330 ± 64 mg/L, 390 ± 16 mg/L, and 219 ± 11 mg/L of β-carotene, respectively (Figure 1C). The accumulation of β-carotene clearly correlated with the level of β-ionone production. This may have been the result of the exogenously introduced *PhCCD1* (K164L) and lck-PhCCD1 increasing the cleavage of β-carotene and an altered carbon flux toward β-carotene synthesis (Daletos et al., 2020). It has also been reported that the introduction of non-oxidative glycolysis pathway and an increased efficiency of the endogenous mevalonate pathway can increase the supply of cytosolic acetyl-CoA and GGPP, driving carbon flow toward carotenoid synthesis (Lu et al., 2020). Compared to the YLBI3118 strain, the YLBI3120 and YLBI3121 strains produced less β-carotene during the first 3 days of incubation, but β-carotene concentration increased significantly after the 6th day (Figure 1C).

To verify the genetic stability of strains YLBI3120, YLBI3121 and YLBI3122, we picked three colonies of each strain following 12-day cultivation on YPDm solid medium and tested these for the presence of the inserted genetic construct using colony-PCR. All the engineered strains contained the inserted DNA sequences, indicating the stability of the created yeast strains (Supplementary Figure 1).

The DCW for the YLBI3120, YLBI3121 and YLBI3122 strain cultures were 36.2 ± 1.5 g/L, 36.2 ± 0.9 g/L and 29.4 ± 0.4 g/L, respectively, corresponding to 146, 152, and 118% of the DCW of the parent YLBI3118 strain (Figure 1D). This was probably because the *Leu* gene was present in the YLBI3120, YLBI3121 and YLBI3122 strains but absent from the YLBI3118 strain (Lu et al., 2020).

To explore the validity of this assumption, we also tested the *Leu* complemented strain YLBI3119 that was constructed in previous experiments (Lu et al., 2020). Under identical culture conditions, the YLBI3119 strain produced higher DCW (33.5 ± 1.8 g/L), while β-ionone (321.8 ± 22.4 g/L) and β-carotene (244.66 ± 35.3 g/L) concentrations were comparable to those seen with the YLBI3118 strain (Supplementary Figure 2). As both the YLBI3120 and YLBI3121 strains showed improved β-ionone production, they were selected for further studies.

### High yield β-ionone production from organic waste

**FIGURE 2:**
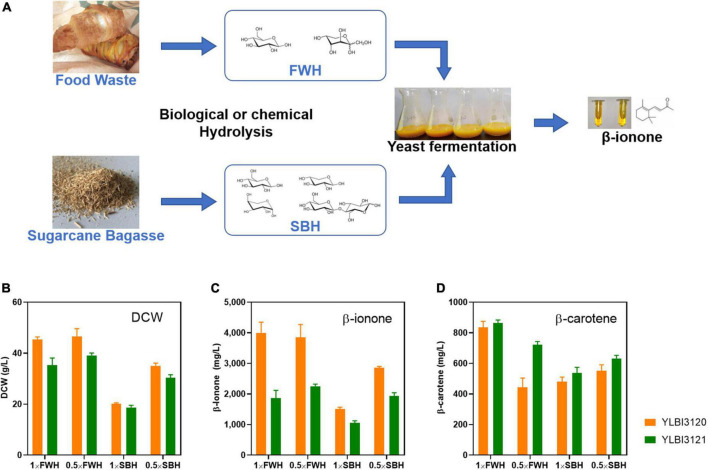
High-yield production of β-ionone from food waste and sugarcane bagasse utilizing an integrated process. **(A)** The integrated process involved two steps. First, the food waste or sugarcane bagasse was converted into simple sugar hydrolysates *via* biological or chemical hydrolysis. These hydrolysates were then fermented with engineered yeast cells to produce β-ionone. Plot shows **(B)** the dry cell weight (DCW), **(C)** the β-ionone concentration, and **(D)** the β-carotene concentration. FWH, food waste hydrolysate; SBH, sugarcane bagasse hydrolysate. Fermentation was conducted with 25 mL of hydrolysate medium containing 10% (v/v) dodecane, and the cultures were incubated with shaking at 250 rpm at 20°C for 12 days. Experiments were performed in triplicate.

Next, we tested the YLBI3120 and YLBI3121 strains for the production of β-ionone from starch-rich food waste and lignocellulose waste, such as sugarcane bagasse. First, these waste materials were hydrolyzed (see section “Biological or chemical hydrolysis of organic waste”) into food waste hydrolysate (FWH) and sugarcane bagasse hydrolysate (SBH) to provide fermentation feedstock (Figure 2A). FWH was produced by the fungal mash hydrolysis method (Yang et al., 2015) and contained 78.4 ± 5.2 g/L of glucose and 8.3 ± 0.6 g/L of fructose. SBH was produced by an alkali-pretreatment and enzymatic hydrolysis (Wang et al., 2020) and contained 34.7 g/L of glucose, 20.1 g/L of xylose, and low concentrations of arabinose and cellobiose (<?2 g/L). These nutrient-rich streams were used directly without any further detoxification, serving as low-cost feedstocks in the production of β-ionone.

Previous studies observed a notable cell-growth inhibition when such feed hyrdolysates were used. The presence of contaminating toxins or the high concentrations of feed compounds were proposed as an explanation for this inhibition (Wang et al., 2020; Wei et al., 2021). Thus, we used both the original hydrolysates (1 × FWH and 1 × SBH) or their diluted forms (0.5 × FWH and 0.5 × SBH) as sole carbon sources for β-ionone production. As shown in Figure 2B, FWH did not appear to inhibit the growth of the YLBI3120 and YLBI3121 strains, whereas SBH inhibited the growth of both *Y. lipolytica* strains tested.

Hydrolysate-mediated inhibition of cell growth and compound production is a typical challenge in the recycling of lignocellulosic waste (Li et al., 2018; Ong et al., 2019; Konzock et al., 2021). Compared to the biomass of YLBI3120 and YLBI3121 strains cultured in YDPm medium (i.e., 35.6–38.8 g/L), the biomass obtained after culturing these strains in 0.5 × SBH medium was slightly lower (i.e., 31.2–35.8 g/L). This suggested that *Y. lipolytica* was relatively tolerant to growth inhibitors in lignocellulosic hydrolysate and could be a promising microbe for generating value-added compounds from organic waste. Similarly, non-detoxified xylose-rich agave bagasse hydrolysate (Niehus et al., 2018) or miscanthus hydrolysate (Yook et al., 2020) have recently been used as feedstock for the efficient production of lipids by *Y. lipolytica*.

Both the YLBI3120 and YLBI3121 strains exhibited an impressive capacity for β-ionone production from FWH and SBH, resulting in significantly higher yields than those achieved in YPDm medium (Figure 2C). The β-ionone concentration after fermenting 0.5 × FWH and 1 × FWH by the YLBI3120 strain was 3.85 ± 0.42 g/L and 4.00 ± 0.35 g/L, respectively, while fermenting 0.5 × SBH or 1 × SBH with this strain resulted in 2.86 ± 0.04 g/L and 1.51 ± 0.06 g/L β-ionone yield. The β-ionone concentrations obtained after fermenting 0.5 × FWH and 1 × FWH using the YLBI3121 strain were 2.25 ± 0.26 g/L and 1.86 ± 0.24 g/L, respectively, while fermenting 0.5 × SBH and 1 × SBH were resulted in 1.93 ± 0.11 g/L and 1.05 ± 0.07 g/L β-ionone yield (Figure 2C). Thus, fermentation of 1 × FWH yielded 1.16–1.35 times more β-ionone than fermenting 1 × SBH. Moreover, the fermentation of 0.5 × FWH yielded 1.76–2.65 times more desired end product compared to fermenting 0.5 × SBH. In addition, fermenting 0.5 × FWH or 1 × FWH with the YLBI3120 strain resulted in the production of 444 ± 59 mg/L or 834 ± 40 mg/L of β-carotene. The corresponding values during the fermentation of 0.5 × and 1 × SBH were 551 ± 39 mg/L and 480 ± 30 mg/L, respectively. Fermenting 0.5 × and 1 × FWH using the YLBI3121 strain accumulated 722 ± 20 mg/L or 864 ± 19 mg/L β-carotene, while 0.5 × and 1 × SBH feedstock resulted in the production of 632 ± 20 mg/L and 537 ± 37 mg/L of β-carotene, respectively (Figure 2D).

Sugar consumption was also monitored during the fermentation (Supplementary Figure 3). Glucose and fructose were depleted during the fermentation of FWH feedstock, while glucose, xylose, and arabinose were all depleted when SBH was fermented. Glucose appeared to be the preferred carbon source. It was depleted after 6 days of fermentation from the 1 × FWH, and after 3 days when fermenting the other three feedstocks (Supplementary Figure 3). These results supported previous observations that glucose caused mild carbon catabolite repression (CCR) on xylose and arabinose in *Y. lipolytica* (Spagnuolo et al., 2018; Ryu and Trinh, 2021). The previously observed inability of the organism to utilize cellobiose was also seen in our experiments (Guo et al., 2015).

These results demonstrated that FWH was a better feedstock than SBH and that YLBI3120 was the optimal strain for the purposes of β-ionone production. Although the DCW and β-ionone production while fermenting SBH were lower, they still far exceeded those seen in YPDm medium (i.e., 1.05–2.86 g/L *vs.* 0.35–0.42 g/L). This is consistent with the fact that the fermentation of nutrient-rich hydrolysates containing mixed sugars results in higher yields than fermenting media where glucose is the sole carbon source (Li et al., 2018; Cho et al., 2020).

## Discussion

Here we report a two-step integrated process for the production of β-ionone from food waste and sugarcane bagasse. This approach achieved a β-ionone yield of 4 g/L, which was significantly higher than previously reported results (Czajka et al., 2018; Zhang et al., 2018; Lu et al., 2020). Furthermore, the rate β-ionone production (13.9 mg/L/h) was also higher than what was achieved in previous studies, e.g., 10 mg/L/h using *E. coli* (Zhang et al., 2018), 2.5 mg/L/h using *S. cerevisiae* (Werner et al., 2019) and 2.7 mg/L/h using *Y. lipolytica* (Czajka et al., 2018). We also believe that the production of β-ionone could be further improved by upscaling and further optimizing the fermentation process. The work presented here demonstrated the potential of *Y. lipolytica* as a platform for the high-yield production of terpenoids. Furthermore, this approach could be adapted to manufacture other valuable terpenoids derived from the MVA pathway, such as paclitaxel (Parayil Kumaran et al., 2010) and astaxanthin (Ma et al., 2021).

Recently, several groups developed various host cells, advanced strategies, and tools for the microbial production of terpenoids (Carsanba et al., 2021; Zhang et al., 2021; Rinaldi et al., 2022). However, as shown in Table 3, only a few studies explored the biorefining of various forms of organic waste to produce terpenoid flavor and fragrance compounds (Table 3; Yao et al., 2020; Zhuang et al., 2020; Styles et al., 2021; Szotkowski et al., 2021; Wei et al., 2021). Moreover, the yields achieved in most of these studies was rather low, usually below 1 g/L. For example, 327 mg/L of ergosterol was obtained from the combination of spent coffee ground hydrolysate combined with coffee oil (Szotkowski et al., 2021), 88.6 mg/L of astaxanthin was generated from sugarcane bagasse hydrolysate (Zhuang et al., 2020), 36.1 mg/L of α-pinene (Wei et al., 2021), and 20.6 mg/limonene (Yao et al., 2020) were obtained from corn stover hydrolysate.

**TABLE 3:** Overview of terpenoid flavor and fragrance compounds production from low-cost feedstocks by microbial cells.

Microorganism	Substrate	Hydrolysis process	Fermentation scale	Product	Titer (mg/L)	Productivity (mg/L/h)	Reference
*Sporidiobolus pararoseus* CCY19-9-6	Spent coffee ground hydrolysate combined with coffee oil	1) acid pretreatment, 2) enzymatic hydrolysis	3.0-L fermenter	Ergosterol	327	4.8	Szotkowski et al. (2021)
*Phaffia rhodozyma mutant* Y1	Sugarcane bagasse hydrolysate	1) alkali pretreatment, 2) detoxification, 3) enzymatic hydrolysis	250-mL shake flask	Astaxanthin	88.6	0.92	Zhuang et al. (2020)
*Parageobacillus thermoglucosidasius MQS6*	Waste bread	NA	250-shake flask	τ-muurolol	14	0.29	Styles et al. (2021)
*Y. lipolytica* YT-31	Lignocellulosic hydrolysate	1) acid pretreatment, 2) detoxification, 3) enzymatic hydrolysis	250-mL shake flask	α-pinene	36.1	0.50	Yao et al. (2020)
*Y. lipolytica* YBX08	Corn stover hydrolysate	1) dry acid pretreatment, 2) detoxification, 3) enzymatic hydrolysis	250 mL shake flask	Limonene	20.6	0.29	Wei et al. (2021)
*Y. lipolytica* YLBI3120	Food waste hydrolysate	1) fungal hydrolysis	250-mL shake flask	β-ionone	3,998	13.88	This study
*Y. lipolytica* YLBI3120	Sugarcane bagasse hydrolysate	1) alkali pretreatment, 2) enzymatic hydrolysis	250-mL shake flask	β-ionone	2,855	9.91	This study

NA, Not applicable.

The highly efficient production of β-ionone reported here relies on genetically engineered *Y. lipolytica* strains overexpressing a variant form of the *PhCCD1* gene. The finding that the *PhCCD1 (K164L)*-containing strain (YLBI3120) produced more β-ionone than the YLBI3121 strain expressing an lck-tagged form of the enzyme, *lck-PhCCD1*, was at variance with previous reports in a different yeast species. In *S. cerevisiae* the overexpression of both of these variants of the enzyme resulted in almost identical β-ionone yields (Werner et al., 2019). The myristoylation of the lck-tagged enzyme modulates its affinity to cell membranes, which in turn influences the activity of PhCCD1. In yeast, protein myristoylation is performed by a myristoyl transferase. While this enzyme is well characterized in *S. cerevisiae* (Duronio et al., 1989) its existence and biochemical characteristics remain to be examined in *Y. lipolytica*. Although we have identified a putative myristoyl transferase in the UniProt database using a basic local alignment search tool [Universal Protein resource (UniProt) No. Q6C7G2], this candidate gene only shows 48.72% identity to the myristoyl transferase (UniProt No. P14743) of *S. cerevisiae* (data not shown). Thus, it is tempting to speculate that the myristoylation of the lck peptide tag may be insufficient in *Y. lipolytica*. This, in turn, could reduce the activity of lck-PhCCD1 fusion protein, affecting the ability of YLBI3121 strain to biosynthesize β-ionone. We expected the chimeric variant of the enzyme containing both the K164L substitution and the lck tag, lck-PhCCD1 (K164L) to show a higher affinity toward cell membrane insertion. Consequently, we hypothesized that the YBLI3122 strain carrying this modified chimeric enzyme would produce better yields than cells overexpressed with either the PhCCD1 (K164L) or lck-PhCCD1. However, in practice, despite the demonstrable overexpression of the inserted *lck-PhCCD1 (K164L)* gene β-ionone yields produced by the YLBI3122 strain were disappointing. This may be attributable to the fact that the active site of the lck-PhCCD1 (K164L) in this fusion protein faces the lipid bilayer, resulting in low CCD activity (Werner et al., 2019).

The cleavage of a β-carotene by CCD1 requires two oxygen molecules to produce two β-ionone molecules. The presence of considerable amounts of β-carotene seen in our experiments suggests that despite oxygen supply in flasks during fermentation may be insufficient, limiting the activity of PhCCD1 (Khadka et al., 2019; Liu et al., 2021). This explanation raises the possibility that both the YLBI3120 and YLBI3121 strains have the potential to produce even higher concentrations of β-ionone under optimal fermentation conditions. Alternatively, β-ionone production could be further improved by additional alterations to the CCD1 enzyme or metabolic engineering aiming to fine-tune the expression of central metabolic, isoprenogenic, and carotenogenic genes.

Although *Y. lipolytica* has endogenous genes for pentose catabolism (Ryu et al., 2016; Ryu and Trinh, 2018) this yeast cannot use xylose as a sole carbon source (Ledesma-Amaro et al., 2016; Niehus et al., 2018; Ong et al., 2019; Prabhu et al., 2020). Conversely, a synergistic effect of mixed-sugar utilization was reported in *Y. lipolytica* (Ryu et al., 2016; Ryu and Trinh, 2021), where xylose could be utilized in the presence of a glucose-xylose mixed sugar feedstock. This observation suggests that the expression of genes involved in xylose catabolism may be activated in the presence of other sugars, including glucose (Tsigie et al., 2011; Ryu et al., 2016; Ong et al., 2019). Recently, both endogenous and heterologous xylose utilization pathways have been introduced into *Y. lipolytica*. The resulting strains promise improved utilization of xylose in the production of chemical compounds and fuel (Wei et al., 2020a,b; Sun et al., 2021). Additional metabolic engineering approaches (Ledesma-Amaro et al., 2016; Rodriguez et al., 2016; Wu et al., 2019), strain mating (Li and Alper, 2020), lab adaptive evolution (Zhou et al., 2020) and the use of artificial chromosomes have all been used to enhance xylose utilization. Although it appears that *Y. lipolytica* utilizes arabinose in a similar way to xylose, only a few studies have investigated arabinose fermentation by this yeast (Tsigie et al., 2011; Ryu and Trinh, 2018, 2021; Spagnuolo et al., 2018). Other groups used either CRISPR tools (Schwartz et al., 2018) or artificial chromosomes (Guo et al., 2020) to introduce the ability of cellobiose degradation and utilization into *Y. lipolytica* cells. These developments raise the possibility of combining the ability to utilize xylose and/or arabinose, and the ability to degrade cellobiose in a single engineered *Y. lipolytica* stain. Such complex modifications of the organism could further enhance the ability of this microbe to produce β-ionone, or other high value compounds, from organic waste.

## Conclusion

Developing an efficient a fermentation process that facilitates the large-scale biosynthesis of flavor and fragrance molecules at several gram per liter concentrations remains challenging. In this study we demonstrated the ability of an engineered *Y. lipolytica*, a GRAS yeast, to produce a commercially valuable aroma compound, β-ionone, from food waste and sugarcane bagasse. Although several aspects of this integrated process remain to be fully optimized, we were able to generate β-ionone at 4 g/L concentration. This is the highest yield achieved to date using the fermentation of organic waste.

This work clearly demonstrates the feasibility of establishing an engineered *Y. lipolytica*-based natural compound supply chain in a biorefinery using inedible food and agricultural waste as feedstock. Our results also reveal that *Y. lipolytica* is a promising host for the biorefining of lignocellulosic waste. Further studies should focus on optimizing culture conditions and the stepwise scale-up of this integrated process from benchtop fermentation to pilot plant scale programs.

## Data availability statement

The original contributions presented in this study are included in the article/Supplementary material, further inquiries can be directed to the corresponding author/s.

## Author contributions

SC: data curation, methodology, validation, formal analysis, investigation, and writing—original draft. YL: methodology, validation, investigation, funding acquisition, and writing—original draft. WW: investigation. YH: writing—review and editing. JW: resources. ST: supervision. CL: supervision, resources, and writing—review and editing. XY: conceptualization, supervision, writing—review and editing, funding acquisition, and project administration. All authors contributed to the article and approved the submitted version.
