# Supplementary material for: Efficient production of the β-ionone aroma compound from organic waste hydrolysates using an engineered Yarrowia lipolytica strain
Source: Front Microbiol. 2022 Sep 21;13:960558. doi: 10.3389/fmicb.2022.960558 (PMC9532697; doi:10.3389/fmicb.2022.960558)
Supplement: Supplementary file 1 [file Data_Sheet_1.docx]

**Supplementary Information**

**Efficient production of the β-ionone aroma compound from organic waste hydrolysates using an engineered Yarrowia lipolytica strain**

Shuyi Chen^1 #^, Yanping Lu^2,5,6 #^, Wen Wang^3^, Yunzi Hu^3^, Jufang Wang^2^, Shixing Tang^1^, Carol Sze Ki Lin^4^ *, Xiaofeng Yang^2^ *

^1^ Guangdong Provincial Key Laboratory of Tropical Disease Research, School of Public Health, Southern Medical University, Guangzhou, Guangdong 510515 China

^2^ School of Biology and Biological Engineering, South China University of Technology, Guangzhou, Guangdong 510006, China

^3^ Guangzhou Institute of Energy Conversion, Chinese Academy of Sciences, Guangzhou, Guangdong 510640, China

^4^ School of Energy and Environment, City University of Hong Kong, Tat Chee Avenue, Kowloon, Hong Kong, China

^5^ Technology Research Center, Wuliangye Yibin Company Limited, 150 Minjiang West Road, Yibin, Sichuang 644007, China

^6^ Postdoctoral Research Workstation, Sichuan Yibin Wuliangye Group Company Limited, 150 Minjiang West Road, Yibin, Sichuan 644007, China

* Correspondence authors:

Email addresses: biyangxf@scut.edu.cn. (X. Yang); carollin@cityu.edu.hk (Carol, S.K. Lin);

^#^ These authors contributed equally to this work.

**Methods for plasmids construction**

**pCAS1yl-POX4**: For the construction of pCAS1yl-POX4, two fragments pCAS1yl-POX4-f1 and pCAS1yl-POX4-f2 were obtained by the amplification from pCAS1yl with primers pCAS1yl-F/gRNA-POX4-R and gRNA-POX4-F/pCAS1yl-R, respectively. The resulted fragments were then assembled to generate pCAS1yl-POX4 by Gibson assembly method. The relative primers are listed in Table S1. The Q5® High-Fidelity DNA Polymerase (NEB) was used for all the PCR amplifications.

**pUC19-POX4-HA:** For the construction of pUC19-POX4-HA, the upstream and downstream homologous arms were amplified from *Y. lipolytica* po1f genomic DNA with the primers POX4-up-NotI-F/POX4-up-R and POX4-down-F/POX4-down-NotI-R, respectively. Then the overlap-extension PCR of the two fragments was performed to generate homologous arm fragment. The pUC19 plasmid was linearized by the amplification with primers pUC19-tong-F/R. The resulted linearized plasmid and homologous arm fragment were then assembled by Gibson assembly method, yielding pUC19-POX4-HA. The relative primers are listed in Table S1.

**pUC19-POX4-Leu2:** Leu2 expressed cassette was amplified from pINA1269 with primers POX4-Leu2-F/R. The pUC19-POX4-HA plasmid was linearized by the amplification with primers pUC19-LEU2-POX4-down-F/pUC19-LEU2-POX4-up-R. The two fragments were then assembled to yield pUC19-POX4up-Leu2-POX4down by Gibson assembly method.

**pUC19-PhCCD1(K164L)**: The pUC19-POX4-Leu2 plasmid was linearized by the amplification with primers POX4-mig1t-F/Leu2-CCD1-R. The PhCCD1 directed mutation expressed cassette was constructed by overlap-PCR with CCD1-Leu2-F/CCD1m-R and CCD1m-F/mig1t-POX4-R from pUC19-P_GPD2_-CCD1-mig1t (Lu et al., 2020). All the three fragments were then assembled to yield pUC19-NotI-POX4up-P_GPD2_-CCD1(K164L)-mig1t-Leu2-POX4down-NotI by Gibson assembly method.

**pUC19-lck-PhCCD1**: The pUC19-POX4-Leu2 plasmid was linearized by the amplification with primers POX4-mig1t-F/Leu2-lck-R. The membrane destination peptides was ligated to the amino-terminal of PhCCD1 by overlap-PCR that amplified with CCD1-Leu2-F/mig1t-POX4-R from pUC19-P_GPD2_-CCD1-mig1t (Lu et al., 2020). All the two fragments were then assembled to yield pUC19-NotI-POX4up-P_GPD2_-lck-CCD1-mig1t-Leu2-POX4down-NotI by Gibson assembly method.

**pUC19-lck- PhCCD1(K164L)**: The pUC19-POX4-Leu2 plasmid was linearized by the amplification with primers POX4-mig1t-F/Leu2-lck-R. The membrane destination peptide was ligated to the amino-terminal of PhCCD1(K164L) by overlap-PCR that amplified with CCD1-Leu2-F/mig1t-POX4-R from pUC19-CCD1m. All the two fragments were then assembled to yield pUC19-NotI-POX4up-P_GPD2_-lck-CCD1m-mig1t-Leu2-POX4down-NotI by Gibson assembly method.

All plasmids containing expression cassettes were sequence-confirmed before transformation into *Y. lipolytica*.


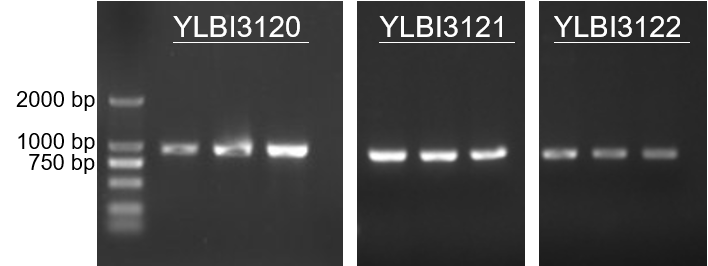


**Fig. S1.** Colony PCR results for confirmed the genotype of strains YLBI3120, YLBI3121 and YLBI3122 after the 12-day cultivation in YPDm medium.


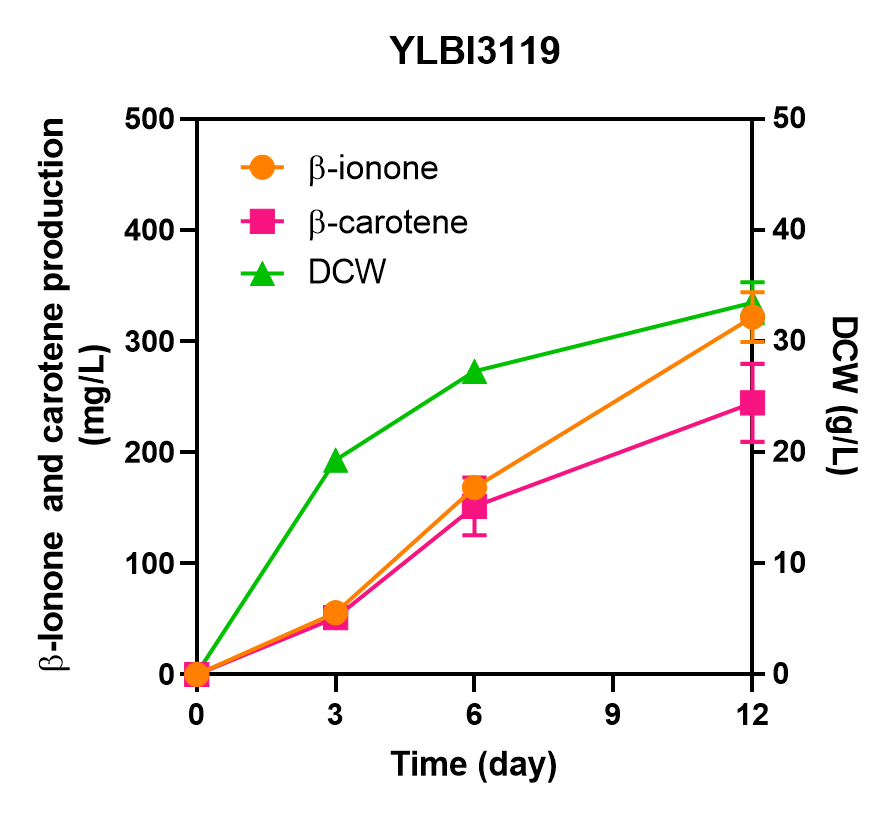


**Fig. S2.** Biomass (dry cell weight, DCW), β-ionone, β-carotene production by strain YLBI3119. Fermentation was conducted with 25 mL of YPDm medium containing 10% (v/v) dodecane, and the cultures were incubated with shaking at 250 rpm at 20 °C for 12 days. Each experiment was performed in three biological replicates.


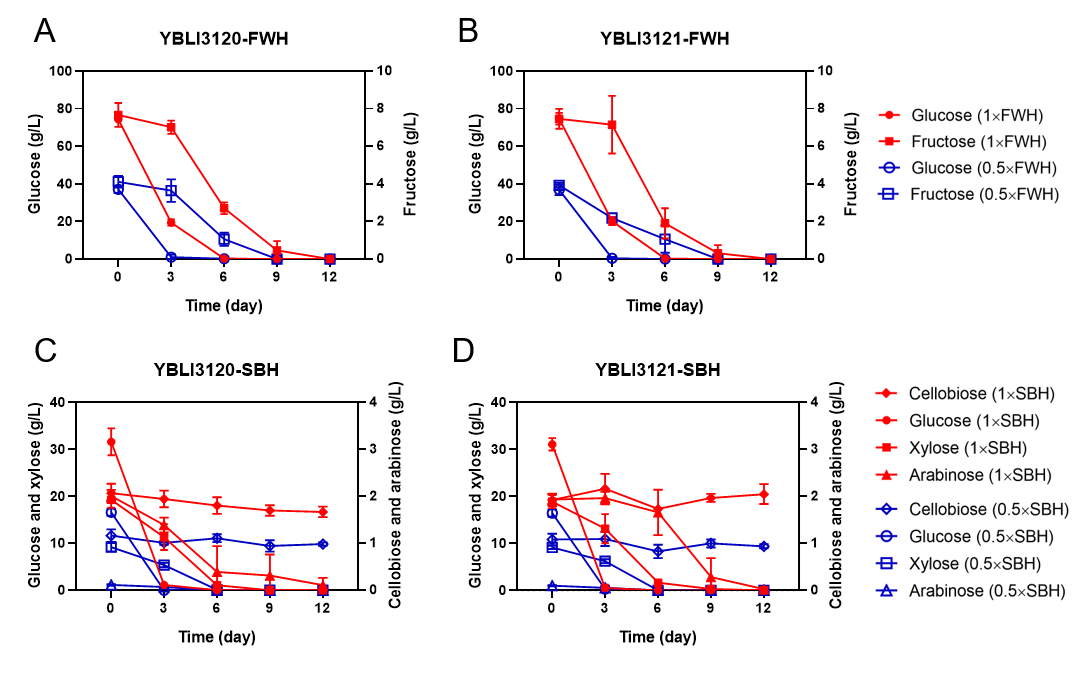


**Fig. S3.** Plots for sugar consumption of YBLI3120 and YBLI3121 in the food waste hydrolysate (FWH) and sugarcane bagasse hydrolysate (SBH). Fermentation was conducted with 25 mL of hydrolysate medium containing 10% (v/v) dodecane, and the cultures were incubated with shaking at 250 rpm at 20 °C for 12 days. Each experiment was performed in three biological replicates.

**Table S1**. Primers used in this study for the construction of plasmids and the confirmation of transformants.

| Name | Sequence (5′ to 3′) |
| --- | --- |
| pCAS1yl-F | TGTGACCGTCTCCGGGAGC |
| pCAS1yl-R | GCTCCCGGAGACGGTCACA |
| gRNA-POX4-F | GAGTTGACGAGAACTGTCGTGTTTTAGAGCTAGAAATAGCA |
| gRNA-POX4-R | ACGACAGTTCTCGTCAACTCGACGAGCTTACTCGTTTCGT |
| Pox4-NotI-up-F | GCTATGACCATGATTACGCCACGCGTGCGGCCGCATATCCTCGGGCTCCATGGGG |
| POX4-up-R | CGCCACCATCCGCAGGAGAGCCCGAAACCCGAC |
| POX4-down-F | TCGGGCTCTCCTGCGGATGGTGGCGTATCTCAGG |
| Pox4-NotI-down-R | AAACGACGGCCAGTGAATTCACGCGTGCGGCCGCCGAAACCCGACTAATTGACTA |
| pUC19-Leu2-POX4-up-R | GCGACGACGGAATTCCGAAACCCGACTAATTGACTA |
| POX4-Leu2-F | ATTAGTCGGGTTTCGGAATTCCGTCGTCGCCTGAGT |
| POX4-Leu2-R | ATTGCCCTGAGATACGAATTCATGTCACACAAACCG |
| pUC19-Leu2-POX4-down-F | GTGTGACATGAATTCGTATCTCAGGGCAATGGTGAG |
| pUC19-pox4d-F | GATGCCCGTGTCCGAGTATCTCAGGGCAATGGTGAG |
| pUC19-pox4u-R | GACTGGAACAGCCCCCGAAACCCGACTAATTGACTA |
| Pox4u-hisG-F | ATTAGTCGGGTTTCGGGGGCTGTTCCAGTCAATCA |
| PTA-xpr2t-pox4-R | ATTGCCCTGAGATACTCGGACACGGGCATCTCACTT |
| Leu2-CCD1-R | CTCCTTTCTACCCATTTTCGGAGGCGATCTGGTCAA |
| CCD1-Leu2-F | AGATCGCCTCCGAAAATGGGTAGAAAGGAGTCTGACGA |
| CCD1m-R | CAGAATCTTCAGCAGCGCTCGCAGCATCTGCATGTA |
| CCD1-m-F | ATGCTGCGAGCGCTGCTGAAGATTCTGGACACCTCGT |
| mig1t-POX4-R | ATTGCCCTGAGATACTTACAGCTTAGCCTGCTCCTGG |
| POX4-mig1t-F | CAGGCTAAGCTGTAAGTATCTCAGGGCAATGGTGAG |
| Leu2-lck-R | GCTCCGGCTCCGGAGTTGGCGGATCGCTCGGGGTTAGACGAACACACACATCCCATTGTTGATGTGTGTTTAATTCA |
| lck-CCD1-F | CCGAGCGATCCGCCAACTCCGGAGCCGGAGCCGGAGCCGGAGCCATCCTGTCCCGAGGTAGAAAGGAGTCTGACGAT |
|  | CGTAGCCGAAGGTGAACATCT |

**Table S2**. Primers used in this study for the confirmation of transformants by colony PCR.

| Name | Sequence (5′ to 3′) |
| --- | --- |
| gRNA-CP-F | TGCAGCTGGCACGACAGGTTT |
| gRNA-POX4-R | ACGACAGTTCTCGTCAACTC |
| lck-CP-F | GAGGCACGCCCTCGAATTTGA |
| lck-CP-R | GTAGTGCAGGGGCTTGGAAGA |
| CCD1-CP-F | ACACCTGCCCGAGTGTCTGAA |
| CCD1-CP-R | CGTAGCCGAAGGTGAACATCT |

**References:**

Lu, Y., Yang, Q., Lin, Z., and Yang, X. (2020). A modular pathway engineering strategy for the high-level production of beta-ionone in *Yarrowia lipolytica*. *Microbial Cell Factories* 19**,** 49.
